# Supplementary material for: Methicillin-Resistant Staphylococcus aureus from Peninsular Malaysian Animal Handlers: Molecular Profile, Antimicrobial Resistance, Immune Evasion Cluster and Genotypic Categorization
Source: Antibiotics (Basel). 2022 Jan 14;11(1):103. doi: 10.3390/antibiotics11010103 (PMC8773339; doi:10.3390/antibiotics11010103)
Supplement: Supplementary file 1 [file antibiotics-11-00103-s001.zip › antibiotics-1513186-supplementary.pdf]

Table S1: Demographic data and molecular profile of MDRSA isolates (n=30).

ID	State	Location ID	Origin	Animals	*S. aureus* strain	AMR genes	Virulence genes	IEC type	*Spa* type
1	Kelantan	1	Animal farmer	Ruminant	MDR-MSSA	*ermA-msrA*	*sak*	N	t095
2	Terengganu	2	Animal farmer	Poultry	MDR-MSSA	*tetK-ermC*	*scn-sak*	E	t4171
3	Terengganu	2	Animal farmer	Poultry	MDR-MSSA	*ermC*	-	N	t4171
4	Pahang	3	Animal farmers	Ruminant	MDR-MSSA	*tetK*	*scn*	H	t189
5	Kuala Lumpur	4	Pet owner	Cats and dogs	MDR-MSSA	*ermC*	*scn-sak*	B	t050
6	Kelantan	5	Veterinarian	Pet and livestock	MDR-MSSA	*tetK-ermC*	-	NT	t9531
7	Kuala Lumpur	6	Veterinarian	Cats and dogs	MDR-MSSA	*tetL-ermC-*	-	NT	t084
8	Johor	7	Animal farmers	Poultry	MDR-MSSA	*tetL-ermA-ermB-ermC-msrA*	*sak*	N	t605
9	Pahang	8	Animal farmers	Poultry	MDR-MSSA	*msrA*	-	NT	t4171
10	Pahang	8	Animal farmers	Poultry	MDR-MSSA	*msrA*	-	NT	t189
11	Pahang	8	Animal farmers	Poultry	MDR-MRSA	*mecA-tetK-tetM-msrA*	-	NT	t189
12	Pahang	8	Animal farmers	Poultry	MDR-MSSA	*tetK-ermA-ermB-ermC-msrA*	-	NT	t189
13	Terengganu	9	Animal farmers	Poultry	MDR-MSSA	*msrA*	-	NT	t2174
14	Terengganu	9	Animal farmers	Poultry	MDR-MSSA	*tetL*	-	NT	t2174
15	Terengganu	9	Animal farmers	Poultry	MDR-MSSA	*tetK*	-	NT	t2174
16	Terengganu	9	Animal farmers	Poultry	MDR-MSSA	*tetL*	-	NT	t127
17	Terengganu	9	Animal farmers	Poultry	MDR-MSSA	-	*sak*	N	t2174
18	Terengganu	10	Animal farmers	Ruminants	MDR-MSSA	*ermA-msrA*	*sak*	N	t050
19	Perak	11	Animal farmers	Aquaculture	MDR-MSSA	-	*sak*	N	t315
20	Penang	12	Animal farmers	Ruminant and poultry	MDR-MSSA	-	-	NT	t189
21	Terengganu	13	Pet owner	Pet	MDR-MSSA	-	*scn-sak*	E	t4171
22	Selangor	14	Pet owner	Pet	MDR-MSSA	*tetK*	*scn-sak-chp*	B	t550
23	Kelantan	15	Animal farmers	Ruminants	MDR-MSSA	-	-	NT	t548
24	Kelantan	16	Animal farmers	Ruminants	MDR-MSSA	-	-	NT	t091
25	Terengganu	17	Animal farmers	Ruminants	MDR-MSSA	*tetK*	-	NT	t714
26	Terengganu	18	Animal farmers	Ruminants	MDR-MSSA	*tetK-ermC-msrA-*	-	NT	t3937
27	Terengganu	19	Animal farmers	Ruminants and poultry	MRSA	*mecA*	*scn-sak*	E	t4171
28	Terengganu	20	Pet owners	Pet	MRSA	*mecA-tetK*	*scn-sak*	E	t3293
29	Terengganu	20	Pet owners	Pet	MRSA	*mecA-tetK*	*scn-sak*	E	t3293
30	Kelantan	16	Animal farmer	Ruminants	MRSA	*mecA*	-	NT	t3080

N= No type; NT= Non-typable
